# Research on the Mechanism and Characteristics of Gel–Microbial Composite Oil Displacement in Hypertonic Heavy Oil Reservoirs

**DOI:** 10.3390/gels11100818

**Published:** 2025-10-12

**Authors:** Baolei Liu, Xiang Li, Hongbo Wang, Xiang Liu

**Affiliations:** 1 School of Petroleum Engineering, Yangtze University, Wuhan 430100, China; 2 State Key Laboratory of Low Carbon Catalysis and Carbon Dioxide Utilization, Yangtze University, Wuhan 430100, China; 3 Research Institute of Exploration and Development, Xinjiang Oilfield Company, PetroChina, Karamay 834000, China; wanghongb@petrochina.com.cn

**Keywords:** gels plugging, microbial enhanced oil recovery, bacillus licheniformis, heavy oil reservoir, cold heavy oil production, enhanced oil recovery

## Abstract

To address the limitations of traditional chemical flooding—such as high cost, environmental impact, and formation damage—and the challenges of standalone microbial flooding—including preferential channeling, microbial loss, and limited sweep efficiency—this study develops a novel composite system for a high-permeability heavy oil reservoir. The system integrates a 3% scleroglucan + 1% phenolic resin gel (ICRG) with Bacillus licheniformis (ZY-1) and a surfactant. Core flooding and two-dimensional physical simulation experiments reveal a synergistic mechanism: The robust and biocompatible ICRG gel effectively plugs dominant flow paths, increasing displacement pressure fourfold to divert subsequent fluids. The injected strain ZY-1 then metabolizes hydrocarbons, producing biosurfactants that reduce oil–water interfacial tension by 61.9% and crude oil viscosity by 65%, thereby enhancing oil mobility. This combined approach of conformance control and enhanced oil displacement resulted in a significant increase in ultimate oil recovery, achieving 15% and 20% in one-dimensional and two-dimensional models, respectively, demonstrating its substantial potential for improving heavy oil production.

## 1. Introduction

With the increasing depletion of global oil resources and the continuous rise in energy demand, heavy oil has become a focal area for future oil and gas development. Statistics indicate that heavy oil constitutes over 70% of the world’s oil reserves. China is rich in heavy oil resources, with estimated reserves of about 26.6 billion tons, indicating substantial development potential. However, due to its high viscosity and poor fluidity, conventional primary and secondary recovery methods exhibit limited effectiveness, making it necessary to employ tertiary oil recovery techniques to enhance production efficiency. Yet, current mainstream technologies such as thermal flooding face issues including high energy consumption and cost, along with risks such as clay swelling in reservoir formations. Although chemical flooding can effectively reduce oil viscosity, it suffers from significant chemical adsorption losses and potential formation damage. In this context, microbial enhanced oil recovery (MEOR) has [1] emerged as a key breakthrough direction for the efficient development of high-permeability heavy oil reservoirs, owing to its environmental benefits, in situ mechanisms, and cost-effectiveness.

At present, microbial enhanced oil recovery has demonstrated promising results. Xianke Chen et al. [2] conducted laboratory experiments under endogenous conditions and screened the strain Bacillus bellus B6, which achieved an emulsification index (EI24) of 100% and a viscosity reduction rate of 97.2%. In terms of field application, Chuanh et al. [3] performed crude oil degradation experiments using two symbiotic oil-degrading bacteria, showing that the combined bacterial consortium could degrade up to 70% of the crude oil. Sokolova et al. [4] screened microorganisms based on sapropel, which produced surfactants and organic acids. These were validated in an industrial pilot at the Tatarstan oilfield, increasing oil production by 5970 tons. However, single-microbial flooding still faces several insurmountable shortcomings in reservoir applications [5]. First, microorganisms require stringent growth conditions and generally survive only in low-salinity and medium-to-low temperature environments. In addition, in high-permeability heavy oil reservoirs, injected microbial fluids tend to migrate along well-developed preferential flow channels formed after water flooding. As a result, the effective sweep range is limited, and stable colonization is difficult to achieve, leaving a significant portion of residual oil untapped. These challenges hinder the large-scale application of standalone microbial flooding in high-permeability heavy oil reservoirs.

**Figure 1:**
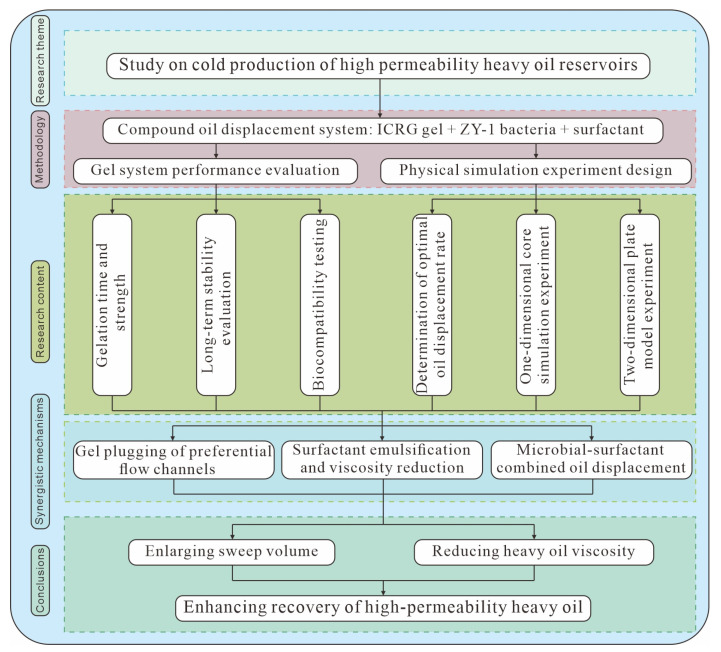
Workflow diagram.

In view of the challenges of low extraction efficiency in high-permeability heavy oil reservoirs and the significant limitations of single tertiary recovery techniques, this study proposes a composite “gel–microbial–surfactant” flooding process. The approach involves screening endogenous emulsifying and biofilm-forming bacteria combined with a biopolymer gel system, followed by co-injection of surfactant and microorganisms into the reservoir. This strategy overcomes the shortcomings of individual tertiary oil recovery methods. By utilizing the metabolic activity of microorganisms, it addresses the inability of gel systems alone to improve crude oil mobility. The process establishes a synergistic mechanism of “blocking + emulsification and viscosity reduction” [6], ultimately breaking through the limitations of single displacement technologies. It thereby enables efficient and environmentally friendly development of high-permeability heavy oil reservoirs and provides technical support for enhancing oilfield production. The workflow of this study is presented in Figure 1.

## 2. Results and Discussion

### 2.1. Screening Results of Oil Displacement by Microorganisms and Their Growth Characteristics

The native microbial community in the formation water was activated. Plate streaking and coating methods were employed to isolate single colonies with crude oil-degrading capability. After successive subculturing, the bacterial suspension was subjected to an NCBI homology analysis, which confirmed the strain as Bacillus licheniformis. The results of the NCBI homology test are presented in Table 1.

Because this strain was extracted from formation water, and it is an endogenous screening culture, it is fully adapted to the reservoir environment. Therefore, there is no need to conduct an environmental adaptability evaluation, and only the growth curve of this strain is determined.

**Figure 2:**
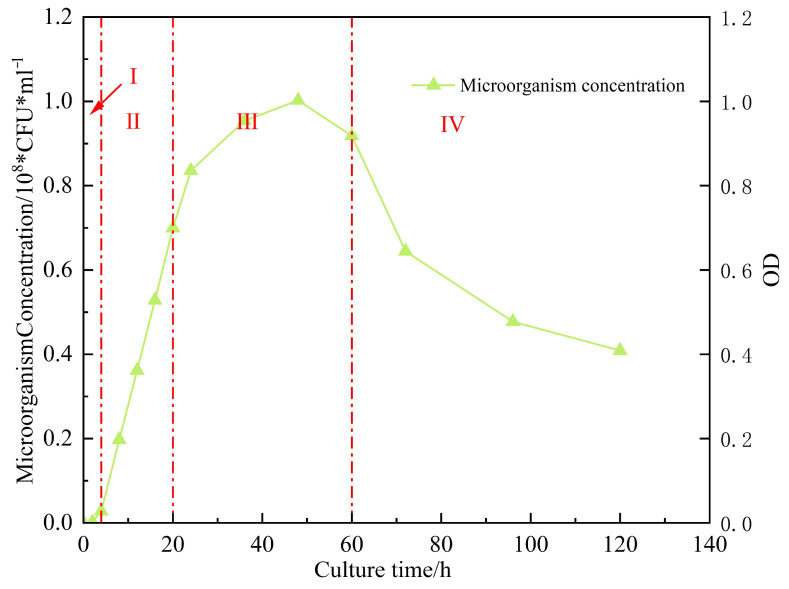
The growth curve of Bacillus licheniformis strains.

The OD_600_ values and microorganism concentration of Bacillus licheniensis in LB liquid medium within 120 h were recorded, respectively, and growth curves were plotted to clarify its growth and metabolic patterns. The results are shown in Figure 2.

Stage I (0–4 h) is the “colonization and adaptation period” of microorganisms in the formation. The OD_600_ value remains between 0.02 and 0.07. This is because [7] the newly transplanted strain needs to adapt to the environment before it can exhibit metabolic functions and synthesize necessary enzymes and metabolic products. At this time, it has not yet entered the stage of massive reproduction, and the number of viable bacteria is basically stable with slow growth. Stage II (4–20 h) is the “efficient action period” for the oil displacement function. The OD_600_ value rapidly rises to 0.76, showing an exponential growth trend. During this stage, the reproduction rate of the strain is the fastest, metabolic activities are vigorous, and a large amount of oil displacement-related products such as surfactants and organic acids are synthesized. In stage III (20–60 h), the OD_600_ value remains stable between 0.76 and 1.02 and rise slowly. At this time, the reproduction rate of the strain is basically the same as the death rate, the number of viable bacteria remains at a relatively high level, and the accumulation of metabolic products reaches the maximum value. Stage IV is the decline period (60–120 h), during which the OD_600_ value slowly drops to 0.455. Without the subsequent addition of nutrients, due to the depletion of nutrients in the culture medium, metabolic waste continuously accumulates. The reproduction rate of the strain is lower than the death rate, but there are still some viable bacteria maintaining basal metabolism. This stage has a relatively small impact on the oil displacement effect, as the metabolic products accumulated in the early stage have completed the emulsification and viscosity reduction of crude oil, and the secondary water flooding can promptly extract the crude oil after the action.

### 2.2. Evaluation of the Functional Characteristics of Bacterial Liquid of Oil Displacement

#### 2.2.1. Emulsification Activity Index of Microbial Fermentation Broth

**Figure 3:**
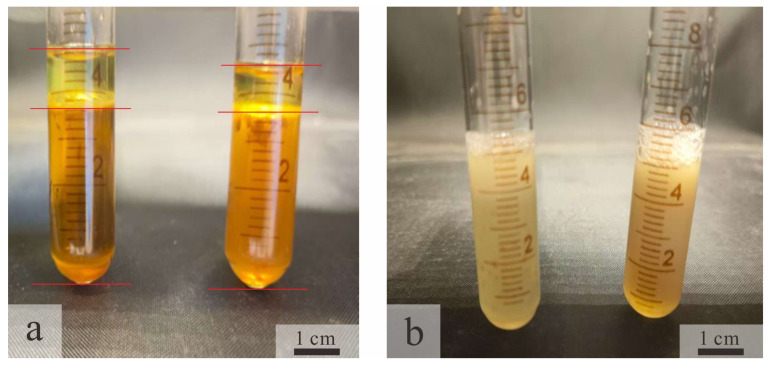
Emulsifying effect of ZY-1 strain (24 h). (**a**) Control group. (**b**) Inoculate Bacillus licheniformis (ZY-1).

The 24 h emulsification index (EI24) is a key metric for evaluating the ability of microbial systems to reduce oil–water interfacial tension and enhance crude oil mobility in microbial enhanced oil recovery. Laboratory results demonstrate that the optimized culture of Bacillus licheniformis achieves an EI24 value of 100% with liquid paraffin, significantly outperforming the uninoculated blank medium (EI24 < 5%), as shown in Figure 3. This high performance is attributed to lipopeptide surfactants produced by bacterial metabolism. These compounds [8] form a stable interfacial film through oriented adsorption at the oil–water interface, effectively preventing emulsion coalescence. As a result, the bacterial broth can persistently disperse crude oil within the reservoir, enhance oil recovery efficiency, and provide essential functional support for field applications.

#### 2.2.2. Evaluation of Emulsification Effect of Microbial Fermentation Broth

**Figure 4:**
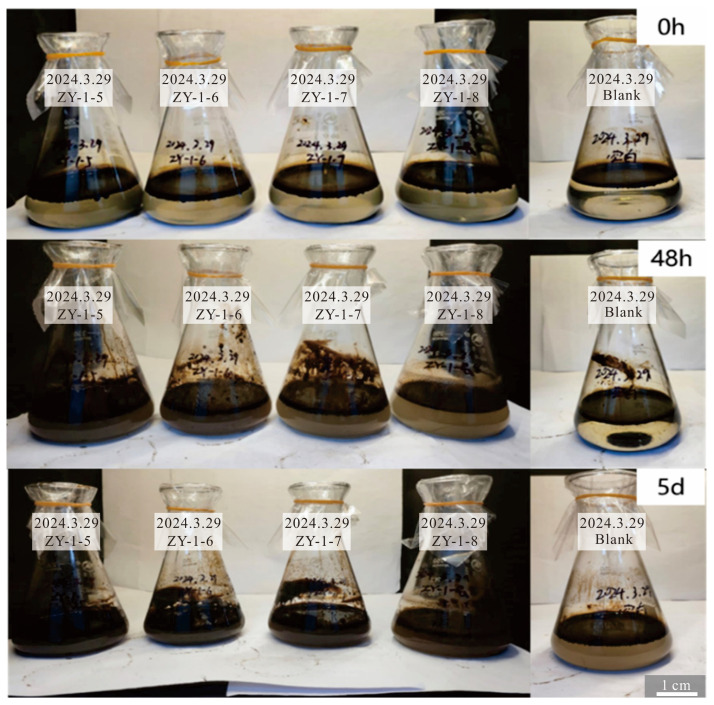
The emulsification effect of ZY-1 bacteria (all on the far right are control groups).

Significant changes in the oil–water phase were directly observed through shake-flask experiments conducted at 50 °C. As shown in Figure 4 (the rightmost sample represents the blank control group), the initial state exhibited clear stratification, with a dark crude oil layer on top and an aqueous phase below. After 48 h of activation by Bacillus licheniformis, the flask contents began to change: the aqueous phase darkened, and the oil phase was no longer aggregated, with small oil droplets appearing and forming a more uniform mixed phase. However, due to the relatively high viscosity of the crude oil, wall adhesion remained noticeable at this stage. Following 5 days of activation and culture [9], the originally distinct oil–water interface largely disappeared, and wall adhesion was markedly reduced. Instead, the oil dispersed into numerous fine droplets uniformly suspended throughout the system, exhibiting typical emulsion characteristics. The mixture showed no rapid stratification even after prolonged standing.

**Figure 5:**
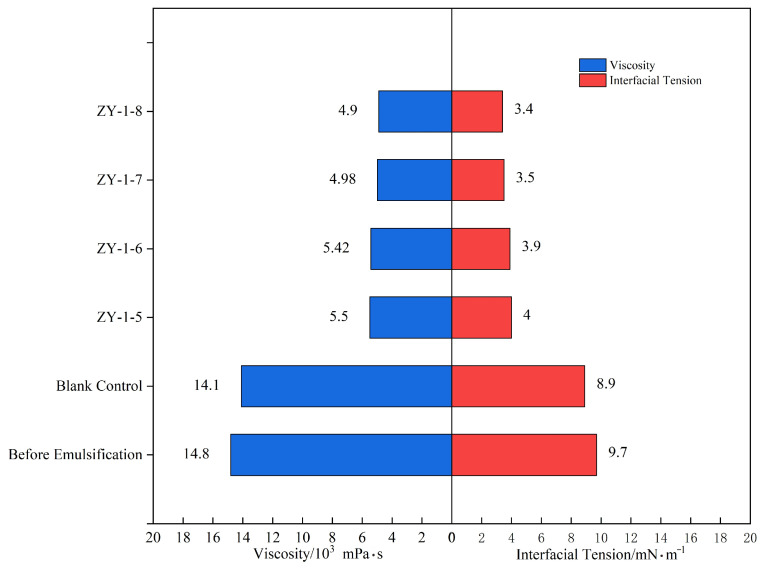
The changes in oil–water interfacial tension and viscosity before and after emulsification.

The interfacial tension between crude oil and water was measured as 9.7 mN/m prior to emulsification. Following treatment with Bacillus licheniformis, the interfacial tension decreased significantly to an average value of 3.7 mN/m, accompanied by a 65% reduction in crude oil viscosity. These results are summarized in Table 2 and illustrated in Figure 5. The marked reduction in interfacial tension provides a critical driving force for crude oil emulsification and dispersion, which aligns with the liquid-phase mixing, oil-droplet refinement, and reduced wall adhesion phenomena observed in shake-flask experiments. Together, these findings confirm the strong crude oil emulsification capability of Bacillus licheniformis.

### 2.3. Test Results of Gel System Performance

#### 2.3.1. Gelation Formation Time and Strength Test

**Figure 6:**
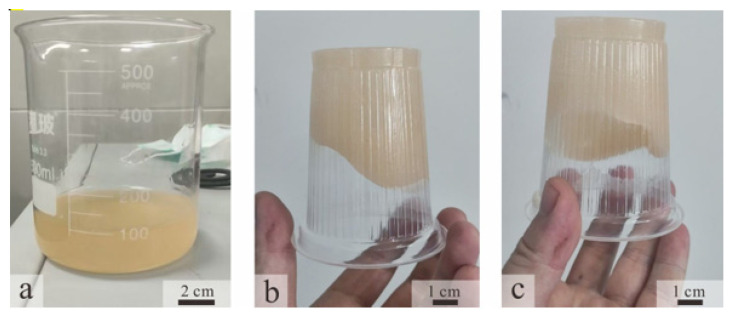
The gelation situation of the scleroglucan + phenolic resin cross-linking system. (**a**) 0 h; (**b**) 24 h; (**c**) 48 h.

Figure 6 illustrates the gel (ICRG 3% scleroglucan and 1% phenolic resin) formation after 24 and 48 h of cross-linking at 50 °C. Evaluated using the visual code method [10], it can be seen that the gel strength has already reached the G grade after 24 h and can reach the H grade after 48 h. The results of ICRG gel-forming time and gel-forming strength under different concentrations of hard glucan are shown in Table 3. It can be seen that with the increase in hard glucan concentration, the strength of the gel after gel formation is also continuously rising. Among them, the strengths of 2% and 3% hard glucan after 48 h of cross-linking reached relatively high levels of G and H grades, respectively.

#### 2.3.2. Evaluation of Gelation Stability

The three ICRG gels were placed in a high-pressure vessel at 50 °C and 10 MPa for 7 days to simulate reservoir conditions. After retrieval, it was found that the three ICRG gels systems still maintained original strength and showed no dehydration phenomenon, demonstrating good stability.

The gel precursor fluid [11] was subjected to high-speed shearing at 6000 rpm using a mechanical stirrer. The viscosity of the base fluid was measured following different shear durations, and the shear degradation rate (D) was quantified based on the percentage of viscosity loss. The calculation method is shown in Equation (1).

D = [(μ_0_ − μ)/μ_0_] × 100% (1)

Among them, μ_0_ the initial viscosity of the gel-based liquid; μ is the viscosity of the gel-based solution after shearing.

**Figure 7:**
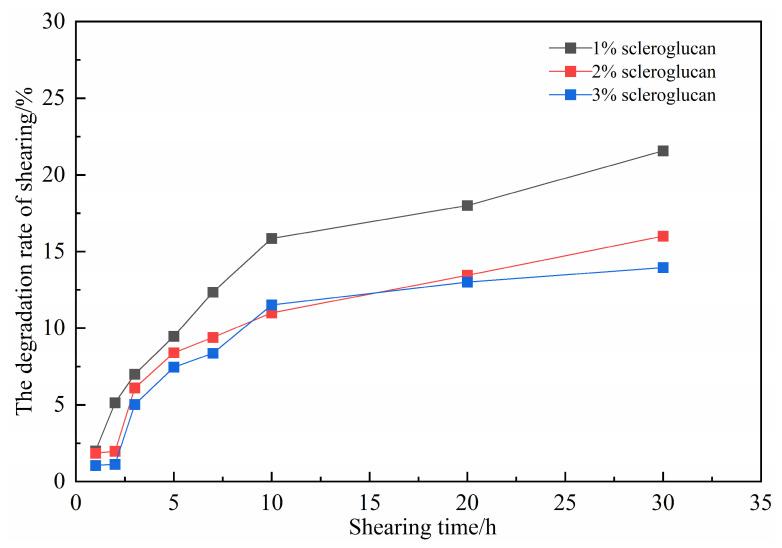
The influence of shear degradation on gel properties.

As shown in Figure 7, the shear degradation rate of the ICRG system increases with the increase in shearing time. As the concentration of the scleroglucan rises, the shear degradation rate of the gel system decreases. After 30 min of shear, the shear degradation rate of the system cross-linked under 3% scleroglucan is only 13.95%. The rigid molecular structure of the system [11,12] confers strong shear resistance, which enables accurate control and prediction of gelation timing after the gel is injected into the wellbore or formation.

Considering the gel formation strength and stability comprehensively, in the following text, 3% scleroglucan +1% phenolic resin cross-linked is selected as the gel system.

#### 2.3.3. Testing Gel–Microbial Compatibility

**Figure 8:**
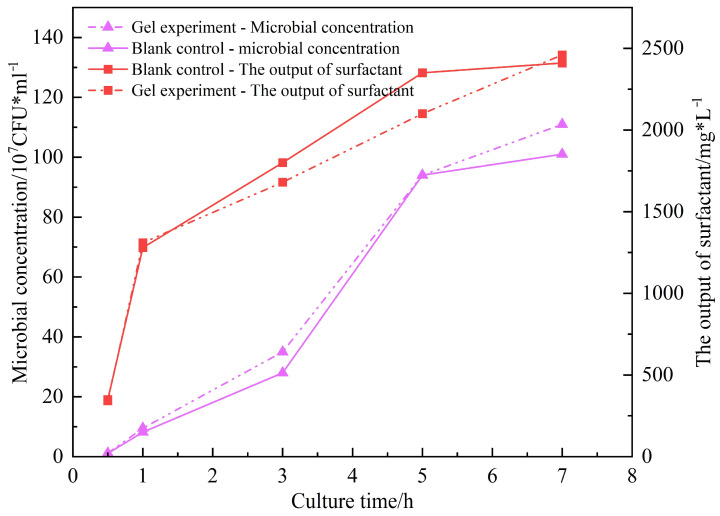
The effect of ICRG gel on the growth and metabolites of ZY-1 strain.

Biocompatibility experiments confirmed that the ICRG gel system exerted no inhibitory effect on the target strain ZY-1 (the screened strain is referred to as ZY-1 hereafter). As shown in Figure 8, the bacterial concentration increased from an initial 1.2 × 10^6^ CFU/mL to 1.01 × 10^8^ CFU/mL during the culture period—slightly higher than that of the blank control group (1.11 × 10^8^ CFU/mL). Meanwhile, the maximum biosurfactant production reached 2460 mg/L, also marginally exceeding the blank group (2410 mg/L). It is hypothesized [13,14] that scleroglucan molecules may act as a supplementary carbon source for microbial metabolism, thereby mildly promoting surfactant synthesis. Throughout the experiment, the gel concentration remained stable, and no adverse effects of Bacillus licheniformis on the gel system were observed. These results confirm the biocompatibility of the scleroglucan-based gel with the microbial system, thereby establishing a foundation for the “gel–microbial–surfactant” composite flooding strategy.

### 2.4. Evaluation of Microbial Oil Displacement Experimental Results

#### 2.4.1. Determining the Optimal Oil Displacement Rate for Microorganisms

**Figure 9:**
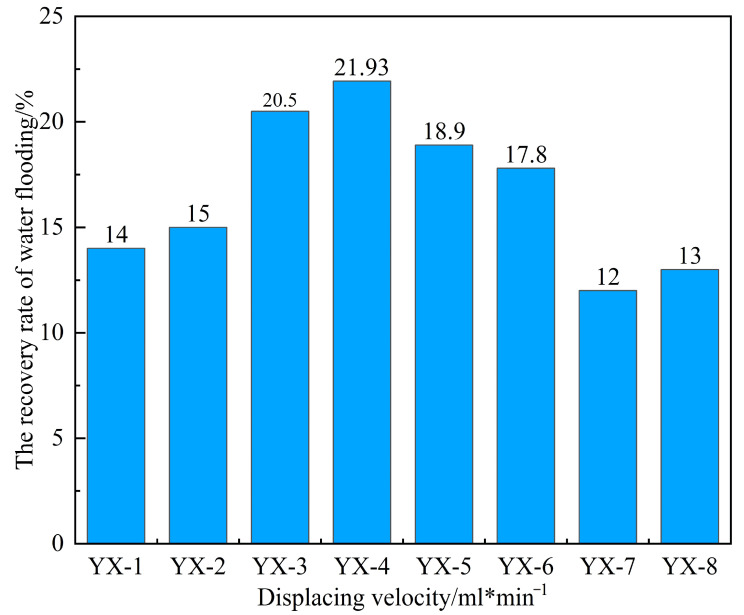
Water flooding recovery rates at different displacement velocities.

**Figure 10:**
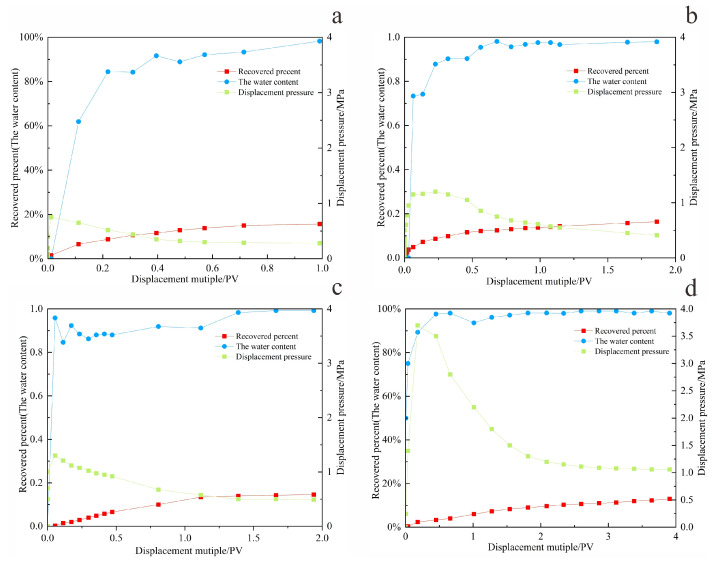
The water displacement process with different displacement speeds. (**a**) 0.1 mL/min; (**b**) 0.3 mL/min; (**c**) 0.5 mL/min; (**d**) 1 mL/min.

By monitoring pressure and flow rate variations during the displacement process under different injection rates, real-time data such as water cut, pressure, and recovery factor were obtained during core flooding, as shown in Figure 9 and Figure 10 and Table 4.

At a low injection rate of 0.1 mL/min (Figure 10a), the water flooding pressure increased gradually and remained at a relatively low level, not exceeding 1 MPa. The water cut rose slowly, and the recovery factor increased at a progressively slower rate with continued pore volumes injected, ultimately reaching an average value of only 14.5%. This is attributed to the high viscosity of the crude oil combined with the low displacement rate, resulting in insufficient driving force. Under these conditions, the aqueous phase fails to effectively mobilize the oil phase, leading to relatively low oil recovery efficiency.

When the displacement rate was increased to 0.3 mL/min (Figure 10b), the injection pressure during the early stage of water flooding rose sharply. After reaching its peak, it decreased gradually while maintaining a relatively high level—neither risking formation fracture due to excessive pressure nor exhibiting inadequate drive energy. The water content initially increased in a fluctuating pattern and stabilized within a suitable range during the steady production stage. The recovery factor continued to rise throughout the experiment, reaching an average of 21.2%, which is significantly higher than that of the low-rate group.

When the displacement rate was further increased to 0.5 mL/min, the injection pressure exhibited a pattern characterized by a sharp initial rise, followed by a precipitous decline, and eventual stabilization. Throughout this process, the water cut increased rapidly, reaching the high water-cut stage within a short period. The oil production rate rose quickly during the early stage of water flooding but then dropped sharply. The resulting recovery factor was slightly lower than that achieved at 0.3 mL/min. When the displacement rate was raised to 1 mL/min, as illustrated in Figure 10d, the excessively high flow rate caused both water cut and injection pressure to increase abruptly during the early displacement stage, indicating significant water channeling. The aqueous phase rapidly established preferential flow pathways, resulting in extensive ineffective water circulation and failure to effectively displace the remaining crude oil. Consequently, the recovery factor at this rate was the lowest among the four displacement rates tested, with an average value of only 12.5%.

**Figure 11:**
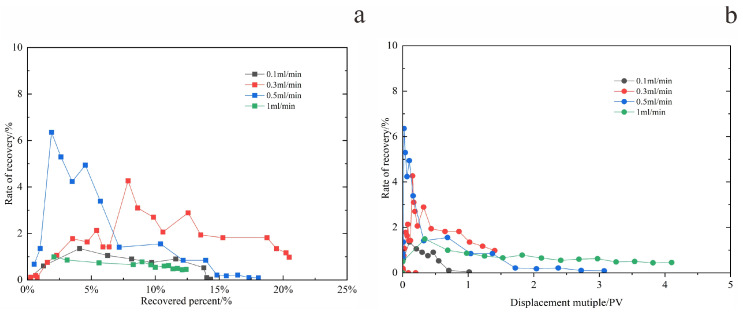
The variation trend of recovery rate with the degree of oil recovery and displacement multiple. (**a**) Recovered percent—oil recovery rate. (**b**) Displacement multiple—oil recovery rate.

As illustrated in Figure 11, which shows the variation in oil production rate with pore volume (PV) injected and recovery factor at two displacement rates, the system with a rate of 0.3 mL/min maintained a relatively high and stable oil production rate throughout the flooding process [15], performing more consistently than that at 0.5 mL/min.

In general, the flow rate governs the initial productivity. However, due to the high viscosity of heavy oil, an excessively high displacement rate can lead to a sharp viscosity increase, promoting “water channeling” through high-permeability zones. Conversely, an overly low rate fails to provide sufficient energy to the reservoir, resulting in poor crude oil utilization. Both scenarios ultimately impair production performance. At the displacement rate of 0.3 mL/min, parameters including pressure, flow rate, water cut, oil production rate, and ultimate recovery collectively reached their optimal balance. Therefore, this rate is identified as the optimum water flooding rate under the experimental conditions of this study.

#### 2.4.2. Experimental Results of Reagent Dosage Optimization

This section systematically evaluates the injection performance of gel, surfactant, and composite systems through core flooding experiments, aiming to provide key insights for the design of subsequent microbial enhanced oil recovery strategies. The experimental results are analyzed as follows.

(1) Selection of gel injection volume

**Figure 12:**
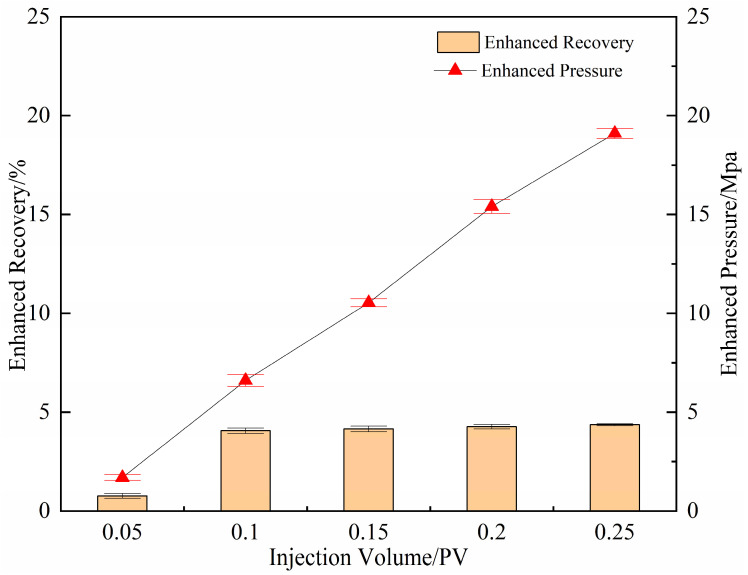
The increase in the maximum pressure and the degree of recovery after gel blocking with different injection amounts.

As shown in Figure 12, the effect of gel injection volume (0.05–0.4 PV) on enhanced oil recovery exhibits a trend of initial increase followed by gradual saturation. When the injected volume was increased from 0.05 PV to 0.1 PV, the incremental oil recovery during subsequent water flooding improved markedly from 0.76% to 4.06% accompanied by an average increase of 5.5 MPa in water flooding pressure. This indicates that a 0.1 PV gel volume is sufficient to establish an effective deep-domain blocking system within the core, which successfully diverts subsequently injected water and expands sweep efficiency.

When the injection volume was further increased from 0.15 PV to 0.25 PV, the incremental recovery showed only a slight rise from 4.06% to 4.36%, with a markedly diminished growth rate. Concurrently, the injection pressure increased sharply, approaching the safe pressure limit of the core and raising the risk of damage formation. Therefore, 0.1 PV was identified as the optimal gel injection volume, offering an effective balance between enhanced oil recovery, operational efficiency, and injecting safety.

(2) Surfactant selection

**Figure 13:**
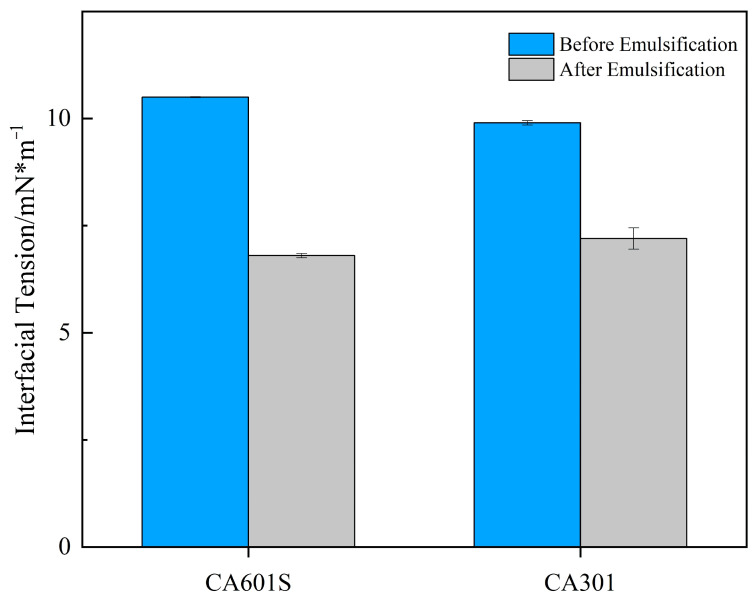
The influence of different surfactants on the interfacial tension between oil and water.

Interfacial tension measurements after 24 h of stabilization (Figure 13) demonstrated that both surfactants significantly reduced oil–water interfacial tension, with CA601S exhibiting comprehensively superior performance. The interfacial tension of the CA601S-treated system decreased from an initial 10.5 mN/m to 6.8 mN/m, a reduction of approximately 40%, whereas the CA301 system decreased from 9.9 mN/m to 7.2 mN/m, a reduction of about 30%. The enhanced performance of CA601S is attributed to its anionic–nonionic molecular structure, which provides improved salt tolerance and interfacial activity. Based on these results, CA601S was selected as the surfactant for subsequent oil displacement experiments in this study.

(3) Optimal injection volume for the oil displacement system

**Figure 14:**
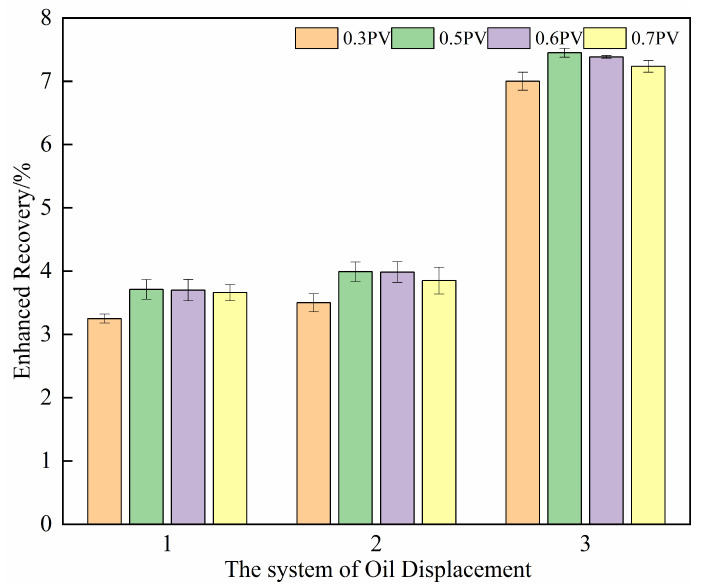
The improvement of the recovery rate in different oil displacement systems with different injection volumes.

The optimization results for the injection volume of the oil displacement system are presented in Figure 14. The incremental recovery initially rises with increasing injection volume but exhibits diminishing returns. As the injection volume increases from 0.3 PV to 0.5 PV, the recovery shows a marked improvement, with an average enhancement of 10%. This is attributed to the sufficient volume ensuring full contact and emulsification action of the composite system in the unswept oil zones.

However, when the injection volume is further increased to 0.6 PV and 0.7 PV, the incremental recovery stabilizes and even declines in Schemes 2 and 3 (as shown in Table 5 and Figure 14). This reduction may result from an “over-injection” effect. In addition, both Schemes 2 and 3 involve microbial growth and metabolism. At higher injection volumes, a portion of the displacing agent may be prematurely produced before microbial degradation and metabolic activity are fully expressed, leading to a lower recovery increase compared to the 0.5 PV case.

In summary, an injection volume of 0.5 PV is sufficient to achieve effective emulsification and significant recovery enhancement. Further increasing the injection volume contributes minimally to ultimate recovery and may even lead to operational inefficiencies. Therefore, 0.5 PV is recommended as the optimal injection volume for the oil displacement system in subsequent field applications.

#### 2.4.3. Experimental Results of the One-Dimensional Model of Microbial Oil Displacement

**Figure 15:**
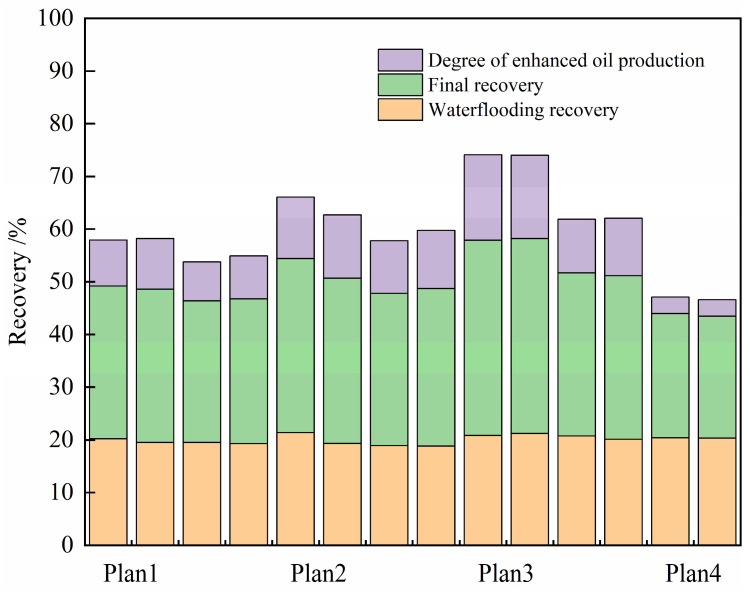
Comparison of the final oil displacement effects of different oil displacement systems.

**Figure 16:**
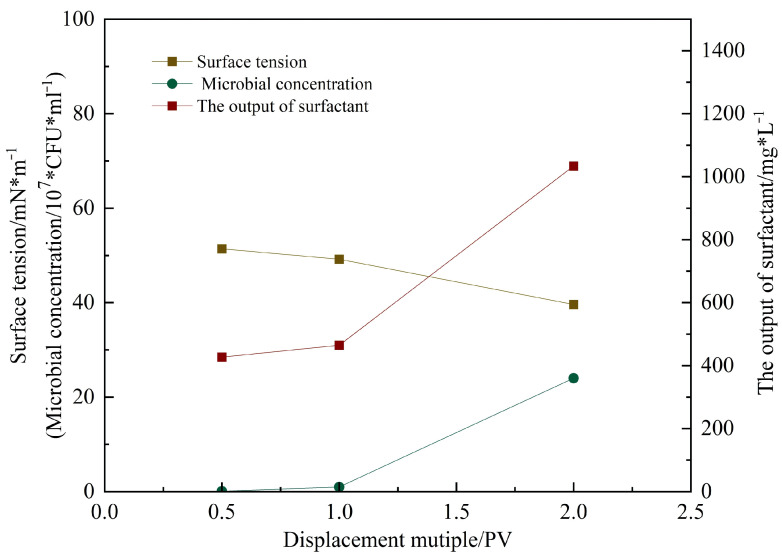
The changes in metabolites, bacterial concentration, and surface tension of the produced liquid after microbial action under different PV.

**Figure 17:**
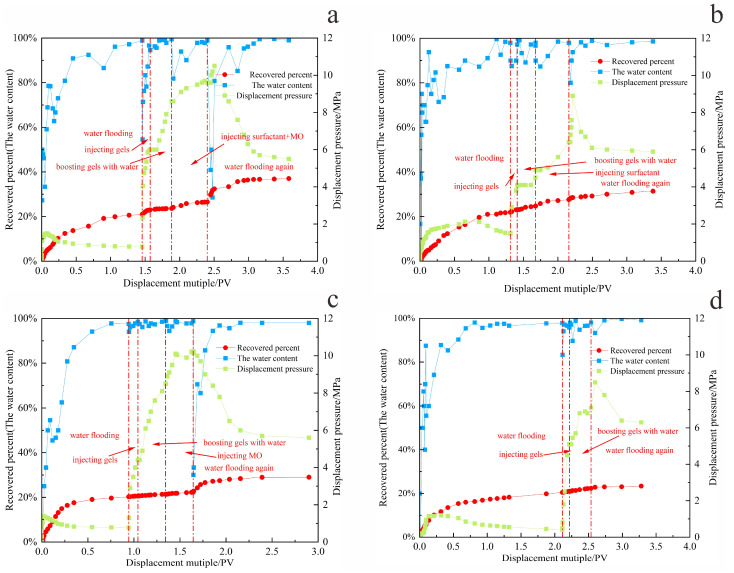
The entire displacement process of different oil displacement systems. (**a**) Mixed injection: surfactant and microorganism; (**b**) injecting only surfactant; (**c**) injecting only microorganism; (**d**) control group.

Based on the displacement rate determined in the previous section, the gel–microbial composite oil displacement experiment was carried out. By comparing the oil displacement effects of four different oil displacement systems, the gel–microbial composite oil displacement mechanism was clarified from the perspective of seepage. The experimental results of different oil displacement systems are shown in Table 6 and Figure 15, Figure 16 and Figure 17.

Figure 15 evaluates the enhancement in oil recovery achieved by different systems. Among all configurations, the co-injection of surfactant and microbial fermentation broth demonstrated the best performance. As shown in Figure 17a, after a 5-day shut-in period, the water cut during subsequent secondary water flooding decreased significantly compared to the initial water flooding stage. Meanwhile, the gel plugging effect maintained sufficient reservoir pressure and improved the sweep efficiency of the displacing fluid.

Figure 16 shows the microbial concentration, surfactant content, and surface tension measured in the produced fluid during flooding at different pore volumes injected [16]. As the injection progressed, the surface tension decreased from 56 mN/m (initial untreated state) to 35.9 mN/m after 2 PV. The surfactant concentration reached 1100 mg/L, and the microbial concentration attained 3.6 × 10^8^ CFU/mL. These results [17] indicate that microbial metabolites rapidly reduce interfacial tension, decreasing capillary and viscous forces, thereby improving crude oil mobility.

Simultaneously, the polymer injected during the conformance control stage plugged the dominant flow channels formed during the initial water flooding, thereby expanding the sweep volume of subsequent flooding agents. This approach not only mobilizes oil in high-permeability zones—otherwise trapped by high viscosity or boundary layer effects—but also enables the displacement of fluid to access previously unswept regions. As a result, microorganisms and surfactants distribute more uniformly within pore spaces, enhancing both the operating efficiency and coverage of the flooding system.

The composite system ultimately achieved a recovery factor significantly higher than other methods, with stable displacement efficiency, delayed water breakthrough, prolonged energy retention, and more complete crude oil extraction. Compared to primary water flooding, the recovery factor was increased by 15%.

The oil displacement performance of the system injected with microorganisms alone is comparable to that of the surfactant-only system. While microbial flooding performs slightly better than surfactant flooding, both are inferior to the three composite systems that utilize synergistic mechanisms. Although microorganisms can alter crude oil properties and partially block high-permeability channels through metabolic activity, the reduction in oil–water interfacial tension by microbes alone remains limited, and their ability to mobilize higher-viscosity crude oil is insufficient, resulting in a relatively narrow swept volume.

Surfactants [17], though effective in reducing oil viscosity and interfacial tension, lack stability and do not provide the pore-scale structural modification and blocking effects offered by microorganisms. Moreover, their emulsifying performance is inferior to that of biosurfactants, and the displacing fluid tends to channel through high-permeability pathways, leading to early water breakthrough. A significant portion of the surfactant is washed out by flowing water before fully contacting the crude oil, which not only reduces chemical utilization but also limits both sweep efficiency and the duration of enhanced oil recovery.

The hybrid injection system effectively combines the advantages of both agents, leading to significantly improved oil recovery. Thus, the recovery factors of the two single-agent systems are considerably lower than those of the synergistic and segmented injection systems. As shown in Figure 17b–d, the system with microorganisms alone maintains low water cut for a longer period compared to surfactant-only injection, extending the duration of the oil enhancement effect by about 5%.

Overall, the synergy between surfactants and microorganisms—whether co-injected or sequentially injected—is key to enhancing oil recovery. Among the tested systems, the co-injection scheme offers real-time synergy and higher efficiency, making it the optimal oil displacement strategy under the experimental conditions.

#### 2.4.4. Research on the Compound Oil Displacement Mechanism of Two-Dimensional Model

**Figure 18:**
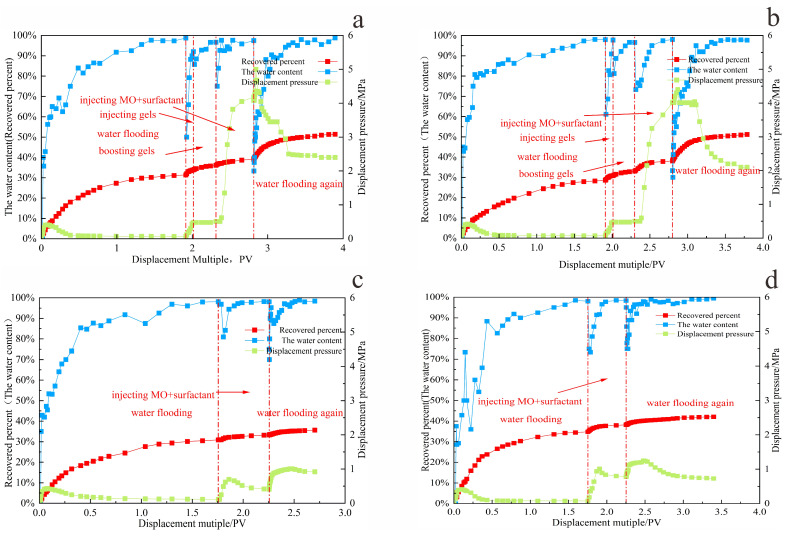
The oil displacement process of the two-dimensional slab model. (**a**) No.A1; (**b**) No.A2; (**c**) No.A3; (**d**) No.A4.

Based on the optimal oil displacement system and injection parameters determined in Section 2.4.1, Section 2.4.2 and Section 2.4.3, the mechanism of gel–microbial composite flooding was investigated using a two-dimensional slab model. As shown in Figure 18a,b, during the gel conformance control stage, the breakthrough pressure in the model increased from 0.4 MPa during water flooding to 4 MPa—an increase of 560%—and remained stable. In contrast, Figure 18c,d depict the process without gel treatment, where the maximum pressure during chemical injection increased by only 0.5 MPa compared to water flooding. In the subsequent water flooding stage after shut-in, the maximum pressure reached 2 MPa, whereas the gel-treated model sustained pressures up to 6 MPa and exhibited a significantly slower water cut rebound.

These results demonstrate that the gel system forms effective physical retention within high-permeability channels, reducing aqueous phase mobility in dominant flow paths. The gel accumulated at the displacement front establishes two uniformly distributed and highly stable sealing zones, diverting the subsequently injected microbe–surfactant system toward unswept crude oil in microchannels. This expands the sweep volume and enhances crude oil recovery.

Furthermore, the gel’s porous structure increases the specific surface area, providing additional attachment sites for microorganisms and prolonging the activity of metabolic products [18]. Amino groups released during gel swelling help buffer formation pH, maintaining an optimal environment for microbial metabolism [6,19]. Gel conformance control also reduces ineffective surfactant loss, enabling more sustained interfacial tension reduction. In turn, organic acids generated by microbial metabolism moderately weaken gel strength, preventing long-term formation damage and establishing a dynamic “blocking–unblocking” equilibrium.

**Figure 19:**
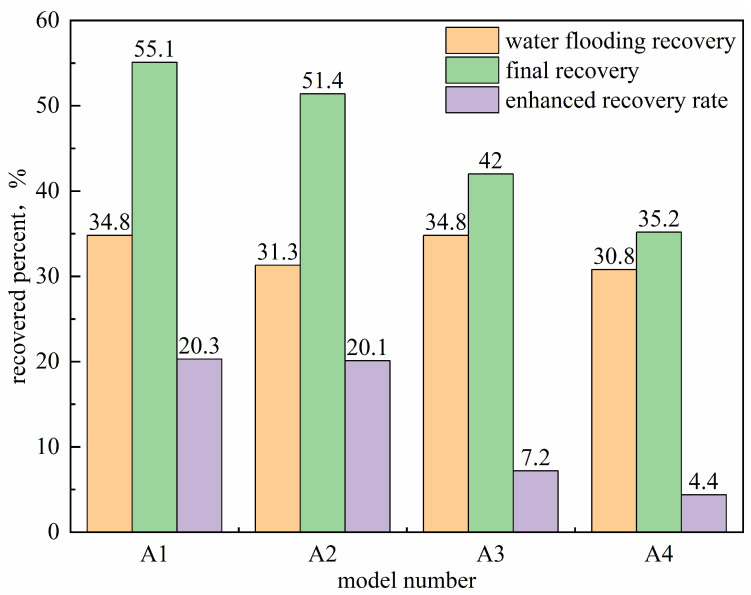
The result of the compound oil displacement of the slab model.

During the subsequent water flooding phase following microbial treatment, the dynamic variations in pressure and water cut reflect clear improvements in displacement efficiency. The initial injection pressure rapidly rose to 5 MPa, then gradually decreased, and stabilized around 2 MPa. This behavior indicates enhanced crude oil mobility due to the combined action of microorganisms and surfactants, while the increased mobilization of previously unswept oil raised overall flow resistance in the porous media. Water cut data show that the breakthrough water cut during the second water flooding was 32%, nearly 40% lower than that of the first water flooding after gel treatment. As shown in Figure 19, the final average crude oil recovery increased by 20%.

These results confirm the synergistic mechanism outlined above: gel-based conformance control expands the sweep region, while microbial degradation and surfactant-mediated viscosity reduction and emulsification collectively lower crude oil flow resistance. This combination enables the effective production of residual oil from previously unswept zones during subsequent water flooding, ultimately enhancing the recovery factor.

**Figure 20:**
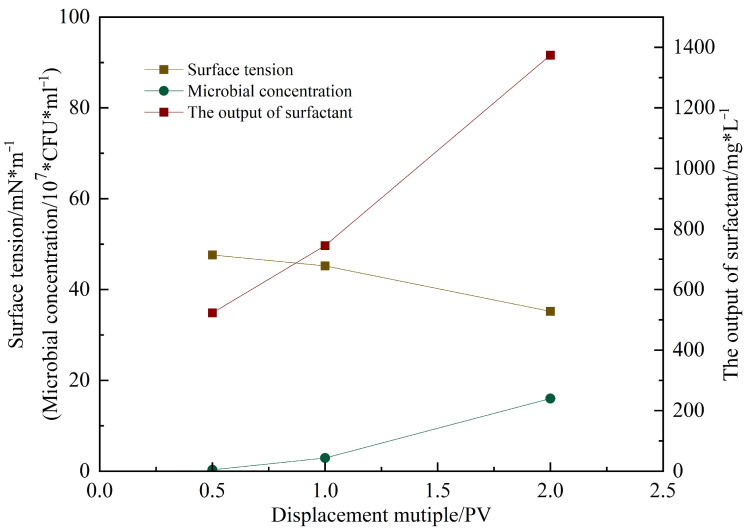
The changes in products, surface tension, and bacterial concentration of the slab model under different PV numbers.

**Figure 21:**
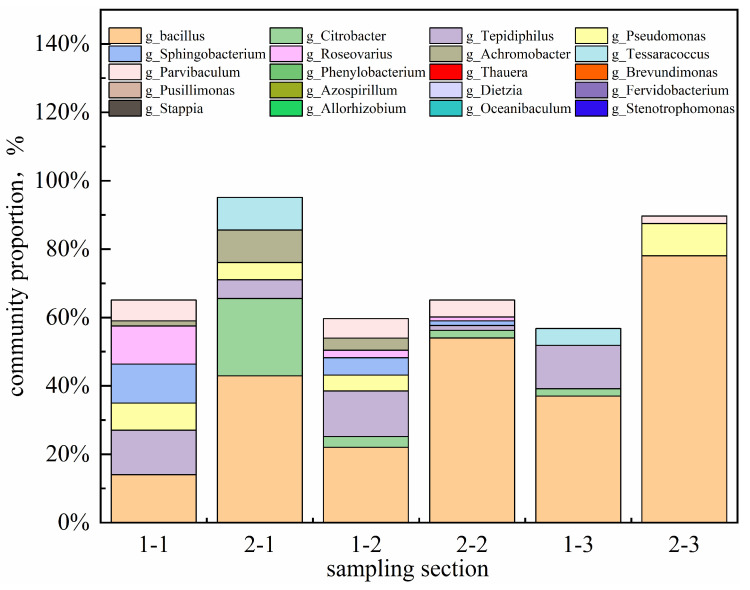
Changes in community structure under different displacement multiples in slab models.

As shown in Figure 20, the microbial concentration during subsequent water flooding increased with injected pore volume (PV), rising from an initial 1.2 × 10^6^ CFU/mL to 2.4 × 10^7^ CFU/mL after 2 PV. Analysis of the produced fluid community structure (Figure 21) revealed a progressive increase in the relative abundance of Bacillus, which exceeded 70% before 2 PV, establishing it as the dominant genus. This indicates successful microbial adaptation and proliferation within the reservoir model. As a hydrophobic bacterium, g_Bacillus possesses strong hydrocarbon-degrading activity [20], providing a functional basis for oil displacement.

Surfactant concentration at the outlet was initially measured at 500 mg/L and continued to increase with further injection, demonstrating continuous in situ production of biosurfactants during microbial metabolism [21]. These biosurfactants function synergistically with the externally injected surfactant to enhance oil mobilization. The hydrophilic carboxyl groups of the surfactants modify rock surface wettability from oil-wet to water-wet, significantly improving residual oil stripping efficiency. Concurrently, the surfactant system substantially reduces oil–water interfacial tension, weakening crude oil adhesion to rock surfaces and facilitating oil mobilization from pore spaces. This dual mechanism promotes the formation of O/W emulsions, effectively reducing crude oil flow resistance through the porous media.

**Figure 22:**
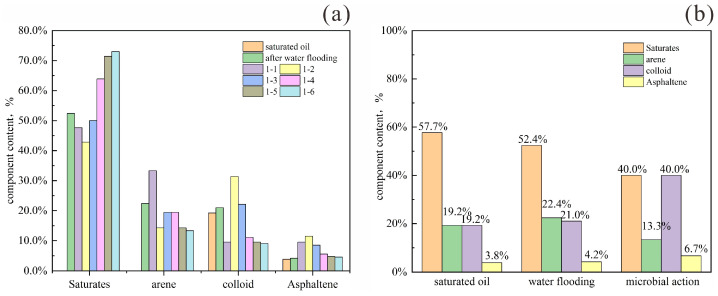
The changes in crude oil components at different stages of gels—microbial oil flooding. (**a**) The changes in crude oil components at different displacement stages in final water flooding; (**b**) the changes in crude oil components at different stages during the oil displacement process.

To track compositional changes during microbial oil recovery, crude oil samples were collected at three stages for component analysis: initial saturation, after water flooding, and after microbial treatment. Results in Figure 22b show that water flooding significantly reduced saturated hydrocarbon content, while increasing the proportions of aromatics, resins, and asphaltenes. This redistribution occurs as water–oil–rock interactions preferentially mobilize less-adsorbed components, leaving polar constituents adsorbed on rock surfaces [22]. Although core heterogeneity influences the precise composition of produced oil, it does not alter the overall four-component trend during water flooding. Pore structure directly affects the adsorption and retention of polar components, thereby altering the composition of produced crude oil.

After microbial treatment, saturated hydrocarbons and aromatics decreased by 12.4% and 9.1%, respectively, indicating preferential microbial degradation of light components (saturates and monocyclic aromatics) [23]. Through β-oxidation, straight-chain alkanes (C_15_–C_30_) are broken down into short-chain fatty acids (e.g., acetic and propionic acid). Using nitrate as an electron acceptor, benzene-series compounds (toluene, xylene) are degraded into CO_2_ and H_2_O, and long-chain hydrocarbons are cleaved into shorter molecules. These processes collectively reduce crude oil viscosity by 65%. The relative increase in resin and asphaltene proportions reflects the consumption of saturates and aromatics. Meanwhile, microbial mild degradation or dispersion of resins and asphaltenes [22,24] prevents the deposition and plugging of heavy components on rock surfaces, further enhancing crude oil mobility.

Figure 22a and Table 7 present the variations in crude oil composition at different displacement multiples during water flooding following microbial treatment. During the medium-to-low water-cut stages (samples 1-1 and 1-2), saturated hydrocarbon content continued to decline, while asphaltene content gradually increased. Aromatic hydrocarbons initially increased and then decreased, whereas resins first decreased and then increased. This pattern arises because production in these stages originates from the front and middle sections of the model, where microbial degradation is most active. Saturated hydrocarbons are preferentially consumed as microbial nutrients, while heavy components including aromatics and asphaltenes are also partially degraded and mobilized.

Beginning with sample 1-3, the system enters the high water-cut stage. Here, the influence of microorganisms and surfactants begins to diminish, and the produced oil shifts toward lighter components, showing an increase in saturates and a decrease in heavy fractions. Aromatic content begins to decline, with saturated hydrocarbons becoming the dominant component.

Upon reaching the ultra-high water-cut stage (samples 1-5 and 1-6), the injected water has largely displaced the original surfactant, and the region affected by microbial degradation has been substantially swept. The produced crude oil at this stage consists predominantly of light components, with saturated hydrocarbons accounting for nearly 70% of the total.

### 2.5. Analysis of Economic and Environmental Benefits

The gel–microbial–surfactant composite cold production technology proposed in this study not only exhibits synergistic mechanisms in its technical approach but also shows considerable potential in economic and environmental benefits. This aligns with the current industrial focus on cost reduction, efficiency improvement, and green, low-carbon transformation.

#### 2.5.1. Economic Benefit Analysis

Compared with traditional thermal methods (e.g., steam flooding) and chemical flooding, the proposed composite technology demonstrates significant potential for cost reduction in the following aspects.

Substantial reduction in energy consumption and operational costs: As a cold production process, the composite system eliminates the need for the substantial fuel (e.g., natural gas), water, and boiler facilities required by thermal methods. By operating under reservoir temperature conditions, it markedly lowers energy input and related operating expenses.

The core oil-displacing microorganisms are screened from native strains and can be fermented using widely available, low-cost feedstocks. Biosurfactants are generated in situ through microbial metabolism, reducing dependence on expensive chemical surfactants and minimizing external chemical requirements. Although the gel system is introduced externally, its relatively low dosage, high stability, and effective plugging performance reduce chemical loss through ineffective cycling and enhance utilization efficiency.

Physical simulation experiments verify that the composite system improves oil recovery by 15% (1D model) and 20% (2D model), respectively. Given the considerable original oil-in-place (OOIP) in heavy oil reservoirs, even a moderate increase in recovery factor can generate significant incremental production and economic returns.

Recent studies focus on further improving economic viability through intelligent regulation of microbial metabolic processes and the development of low-cost, high-performance gel systems. For example, optimizing nutrient injection strategies can selectively activate functional microorganisms, minimize nutrient waste, and sustainably lower operational costs.

#### 2.5.2. Environmental Benefit Analysis

The environmental sustainability of this technology represents one of its core advantages, actively supporting the green development objectives of the oil and gas industry.

Greener Reservoir Fluids: The scleroglucan gel used demonstrates excellent biocompatibility and biodegradability. Biosurfactants and organic acids generated through microbial metabolism are more readily degraded in natural environments compared to synthetic chemical alternatives, preventing secondary pollution during reservoir flow and produced fluid treatment. This significantly reduces subsequent processing challenges and costs, thereby minimizing the overall environmental footprint.

Reduced Formation Damage and Resource Waste: The synergistic interaction between the gel and microorganisms establishes a dynamic “plugging–unplugging” equilibrium, avoiding permanent reservoir damage typically caused by aggressive chemicals or high-temperature operations. This supports the long-term sustainable development of oilfields. By expanding sweep efficiency, the technology enables more thorough mobilization of subsurface resources, reduces residual oil retention, and aligns with the principles of a resource-efficient society.

In recent years, the growing integration of Environmental, Social, and Governance (ESG) concepts in the energy industry has increased the preference for extraction technologies that minimize ecological impact. The approach developed in this study effectively integrates enhanced oil recovery with environmentally conscious production, offering a competitive technical pathway for the sustainable development of high-permeability heavy oil reservoirs.

In summary, the gel–microbial–surfactant composite cold production technology is not only technically and economically viable but also exhibits outstanding environmental performance, positioning it as a promising green and efficient enhanced oil recovery solution.

## 3. Conclusions

This study systematically validates the effectiveness of a gel–microbial composite flooding system for enhanced oil recovery in high-permeability (>100 mD) heavy oil reservoirs. The core innovation lies in the synergistic integration of an in situ cross-linked gel (ICRG) and the indigenous microbial strain ZY-1, which collectively address the key limitation of poor fluid diversion in conventional microbial flooding. The main findings and conclusions are summarized as follows:

The developed ICRG system (3% scleroglucan + 1% phenolic resin) exhibits outstanding performance under reservoir conditions (50 °C), achieving high gelation strength (Grade G in 24 h, Grade H in 48 h), excellent long-term stability (maintained for 7 days at 10 MPa), and low shear degradation (13.95% after high-speed shearing). Importantly, the gel shows full biocompatibility with strain ZY-1.

The composite system functions via a clearly established synergistic mechanism: The ICRG gel physically plugs high-permeability channels through its robust three-dimensional network, effectively diverting subsequent fluids into previously unswept oil-bearing zones. Its porous structure also serves as an adhesion carrier for microbial colonization and sustained activity.

The ZY-1 strain, effectively directed into unswept areas, plays a critical role in mobilizing residual oil. It substantially modifies crude oil properties by degrading long-chain saturates (12.4% reduction) and monocyclic aromatics (9.1% reduction) through β-oxidation, further reducing viscosity. Metabolized biosurfactants (interfacial tension reduced by 61.9%) synergize with injected surfactants to form stable O/W emulsions and weaken crude oil adhesion to rock surfaces.

The combined effect of gel-based conformance control and microbial oil component modification significantly enhances oil recovery (up to 20% OOIP), extends the low water-cut production period, and offers a more efficient and environmentally sustainable alternative to traditional chemical flooding methods.

A key limitation of this work is the absence of direct visual evidence regarding in situ microbial transport, colonization, and pore-scale oil displacement dynamics within opaque porous media. Future studies should employ advanced visualization techniques—such as microfluidics and real-time imaging—to directly observe these fundamental processes. This will further clarify the dynamic interactions within the composite system and support the optimization of field implementation strategies in heterogeneous reservoirs.

## 4. Materials and Methods

### 4.1. Main Reagents and Equipment

Crude oil seed culture medium: 6% crude oil, 1% sodium nitrate, 0.5% disodium hydrogen phosphate, and 0.5% sucrose.

LB medium (pH = 7): 1% peptone, 0.5% sodium chloride, 0.5% beef extract, and (AGAR).

Organic reagents: n-hexane, dichloromethane, methanol, scleroglucan, phenolic resin, and surfactant CA601S.

Experimental instruments and equipment: ultraviolet spectrophotometer, intermediate container, gas flowmeter, core holder, high-temperature and high-pressure reactor, test tubes, valves, shake flaps, plates, collection bottles, gel-strength tester, rheometer, interfacial tension meter, viscometer, artificial cementation core (2.5 × 30 cm), and two-dimensional plate model (30 × 5 × 1 cm).

### 4.2. Sample Collection

Oil and water samples were aseptically collected from a producing well in a target oilfield using sterile containers. All experimental measurements were conducted at 50 °C to simulate reservoir conditions, unless otherwise specified.

### 4.3. Screening of Viscose-Reducing Microorganisms

Oil and water samples were used to prepare a crude oil-based seed culture medium to activate emulsifying and biofilm-forming bacteria in the formation water. The medium was incubated on a shaker at 140 r/min and 37 °C for 7 days. After cultivation, the fermentation broth was serially diluted (10^−1^, 10^−2^, …), and 100 μL of each dilution was spread onto solid LB agar plates. Colonies exhibiting smooth, white morphology and larger oil-clearing zones were selected as candidate strains. These strains were subsequently purified using the streak plate method. Isolated single colonies were inoculated into LB medium and cultured for 24 h. Finally, the taxonomic identity and genetic characteristics of the screened strains were determined through the NCBI homology analysis.

### 4.4. Evaluation of Strain Performance

#### 4.4.1. Determination of Emulsification Activity Index

Liquid paraffin was employed as a model crude oil substitute. In accordance with the method described in [2], 1 mL of liquid paraffin and 4 mL of bacterial supernatant (fermentation broth) were added to a 5 mL test tube. After initial visual measurement of the liquid and organic phase heights, the mixture was vortexed thoroughly and allowed to stand statically at 37 °C for 24 h. The height of the resulting white emulsion layer was then recorded. The 24 h emulsification index (EI24) was calculated as follows:

EI24 = (height of emulsion layer/total height of organic phase) × 100%

#### 4.4.2. Evaluation of Emulsification Effect

Crude oil from the block well group was used to set up one experimental group, three parallel experimental groups, and one blank control group. For each group, 100 mL of crude oil seed culture medium was added to 250 mL conical flasks, and 5% emulsifying bacteria fermentation liquid was inoculated. The flasks were then placed in a water bath shaker at 50 °C and 200 r/min for 5 days. Photos were taken every 24 h to record the emulsification of crude oil in the conical flasks. Five days later, the emulsified shake-flask solution and fresh oil–water samples were, respectively, taken for interfacial tension measurement.

#### 4.4.3. Determination of the Strain’s Growth Curve

Briefly, 2% emulsified bacteria fermentation liquid was taken, inoculated into the LB liquid medium, and incubated in a shaker at 37 °C and 140 r/min for 20 h (the incubation time is the growth logarithm period). Then, several 10 mL sterilized test tubes were taken and marked as 0 h, 2 h, 4 h, etc. After 48 h, 5 mL of LB liquid medium was dropped into each test tube, and 2% of the cultured strain fermentation liquid was added. The mixtures were gently shaken, and the culture was maintained under the same conditions. According to the marked time points, the corresponding test tubes were taken out on time for subsequent OD value determination. At the same time, bacterial counting was carried out by the dilution coating method. The samples were diluted in a series of tenfold gradients with sterile physiological saline. Then, 0.1 mL of each appropriately diluted bacterial solution was selected and dropped onto solid AGAR plates, respectively. Finally, they were evenly coated with sterile coating sticks. After inverting the plates and incubating them at a constant temperature for 24 h, plates containing 30 to 300 single colonies were selected for counting. Then, the colony-forming units of the original sample were calculated according to Equation (2) during the determination.

*C* = *N* × 10*^M^* (2)

Here, *C* represents the microbial concentration, *N* is the number of colonies on the plate, and *M* is the multiple of dilution coating.

The wavelength was set to 600 nm, and the uninoculated sterile liquid culture medium was used as the blank control. After calibration, the bacterial liquid to be tested was thoroughly shaken, and then added to a cuvette to measure its OD value. (Each sample was tested at least three times, and the average value was recorded.) A growth curve was then plotted using culture time as the abscissa and OD value (or the logarithm of the viable bacteria count) as the ordinate to analyze the characteristics of the growth curve.

### 4.5. Preparation and Evaluation of the Performance of Gel Systems

In heavy oil reservoirs, the flow of the aqueous phase is relatively difficult. Thus, during subsequent injection into the oil displacement system, dominant channels for the aqueous phase are already formed in the later stages of water flooding, and so without any treatment, the injection agents will continue to flow through these dominant channels, resulting in an affected range of the oil displacement system that is too small. In response to the above requirements, it is necessary to consider choosing a gel with good biocompatibility. At the same time, its sealing strength needs to be moderate. It should neither completely block the original dominant channel nor cause the sealing layer to be broken through by the displacement phase in the subsequent displacement stage, leading to water seepage. According to relevant data, the gel system composed of scleroglucan and phenolic resin cross-linked (ICRG will be used to refer to this system in the subsequent articles) was selected for experiments [25].

#### 4.5.1. Gel Formation Time and Gel Formation Strength Test

The visual inspection method was adopted for the determination. The prepared gel-based solution was placed in a constant temperature box at 50 °C, and the gel-forming process was recorded every 24 h. After the gel had completely formed, the container was turned upside down to observe and evaluate the strength of the gel system.

#### 4.5.2. Long-Term Stability Evaluation of Gel

After gelation, the gel was placed in a high-temperature autoclave (50 °C, 10 MPa), and samples were taken at 1 day, 3 days, 5 days, and 7 days to [26,27] determine the strength retention rate. The shear degradation rate was evaluated after shearing at 6000 r/min for 30 min.

#### 4.5.3. Gel Biocompatibility Test

The gel was mixed with the Bacillus licheniformis (ZY-1) strain at a volume ratio of 1:1 and co-cultured on a shaker at 50 ° C and 140 r/min for 7 days. Samples were taken at 1 day, 3 days, 5 days, and 7 days, and changes in bacterial concentration at different times were recorded by the LB plate coating counting method. Taking the gel-free bacterial liquid culture system as the blank control, the effect of gel on the growth and metabolism of microorganisms was evaluated.

### 4.6. Gel–Microbial Oil Displacement Physical Simulation Experiment

#### 4.6.1. Interfacial Tension Measurement

At 50 °C, the interfacial tension between the oil phase and the water phase was measured by the pendant drop method using an interfacial tensiometer. To ensure accuracy, both phases were subjected to ultrasonic degassing before the test. During testing, a precision syringe was used to slowly form a suspended water droplet in a transparent sample cell filled with the oil phase. Then, a stable and clear image of this droplet was captured using a high-speed camera, and the contour of the droplet was fitted with the Young–Laplace equation to accurately calculate the interfacial tension between oil and water.

#### 4.6.2. Selection of Oil Displacement Speed

A high-permeability (Kg = 800 mD) artificial rock core (2.5 × 30 cm) was dried for 24 h and weighed, and its gas permeability was measured using a gas flowmeter. Then, the core was vacuumed for 24 h and saturated with the formation water. After [28,29] saturation, the wet weight of the core was recorded using an electronic balance. The difference between dry and wet weights was used to calculate the pore volume of the core. The water-saturated core was then subjected to 20 PV of water flooding. After the pressure stabilized, the liquid permeability was measured. Next, for the water-saturated core, oil saturation was performed by placing the core in a core holder under a confining pressure of 5 Mpa and in a 50 °C constant temperature box. Crude oil was injected at a rate of 0.1 mL/min until no water was discharged from the outlet, indicating saturation. The saturated oil volume was recorded. After aging the core for 7 days, water flooding was carried out at different speeds (0.1, 0.3, 0.5, 1 mL/min) until the moisture content at the outlet reached 98%. The pressure and flow rate during the water flooding process were recorded. The degree of recovery and changes in oil recovery rate at different displacement speeds were compared to determine the optimal displacement rate.

#### 4.6.3. Optimal Dosage of Injection Agent for the Subsequent Oil Displacement Process

(1) Gel injection volume optimization experiment

Five injection volume gradients of 0.05 PV, 0.1 PV, 0.15 PV, 0.2 PV and 0.25 PV were selected. After water flooding, gels with different PV numbers were injected into the cores. After completion of injection, another stage of water flooding was carried out to push the gel towards the middle position of the core, followed by a 1-day waiting period for solidification. After 1 day, water flooding was carried out again, and both the improvement in the extraction degree and the pressure changes during the water flooding process were recorded.

(2) Optimization experiment of Chemical Surfactants

Based on the on-site performance in the oilfield block, where the two surfactants CA601S and CA301 were evaluated for their emulsifying effects, further optimization was carried out: The two surfactant solutions were prepared by diluting them to the specified concentration (50%) using formation water. They were then mixed with crude oil at a volume of 1:1 and poured into a shake-flask at a ratio of 1. The mixture was shaken well and allowed to stand for 24 h. The change in the interfacial tension between oil and water before and after emulsification was measured. At the same concentration, the more the interfacial tension decreases, the better the emulsification effect of the surfactant.

(3) Optimal injection volume for the oil displacement system

Four injection volume gradients of 0.3, 0.5, 0.6, and 0.7 PV were selected. After water flooding, the oil displacement agent was injected into the core according to the injection schemes outlined in Table 8. The two ends of the core holder were closed, and the core was incubated at a constant temperature of 50 °C for 5 days. Then, water flooding was carried out again until the water content reached 98%, and the improvement in the recovery rate in the subsequent water flooding stage was observed.

#### 4.6.4. One-Dimensional Core Model Oil Displacement Experiment

**Figure 23:**
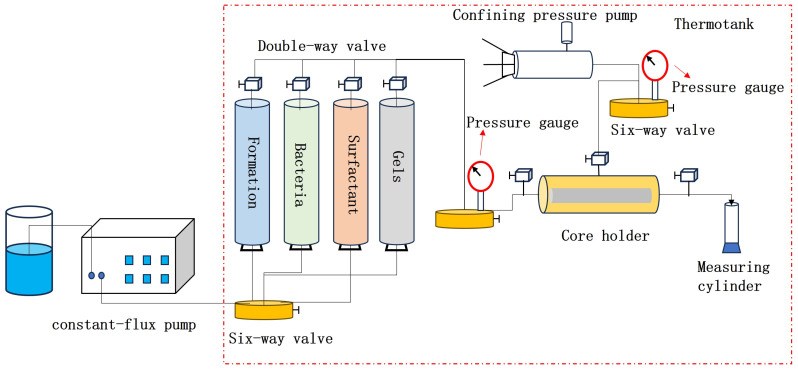
Schematic diagram of the one-dimensional model experimental setup.

Based on the experimental results from Section 4.6.2, 0.1 PV of the ICRG gel system was first injected into the core after water flooding, and 0.3 PV of formation water was injected into the middle section of the model to promote coagulation. One day later, different combined systems were injected at the optimized displacement rate (shown in Table 5). After completion, both ends of the core were closed. The core was then incubated at a constant temperature for 5 days and water flooding was performed again until the water content reached 98%. The pressure and flow rate during the water flooding process were recorded, and samples of the effluent at different flooding ratios were also collected. Next, the microbial metabolites, community structure, and bacterial concentration of the water samples were analyzed. Figure 23 shows a schematic diagram of the oil displacement device.

#### 4.6.5. Research on the Compound Oil Displacement Mechanism of the Two-Dimensional Model

**Figure 24:**
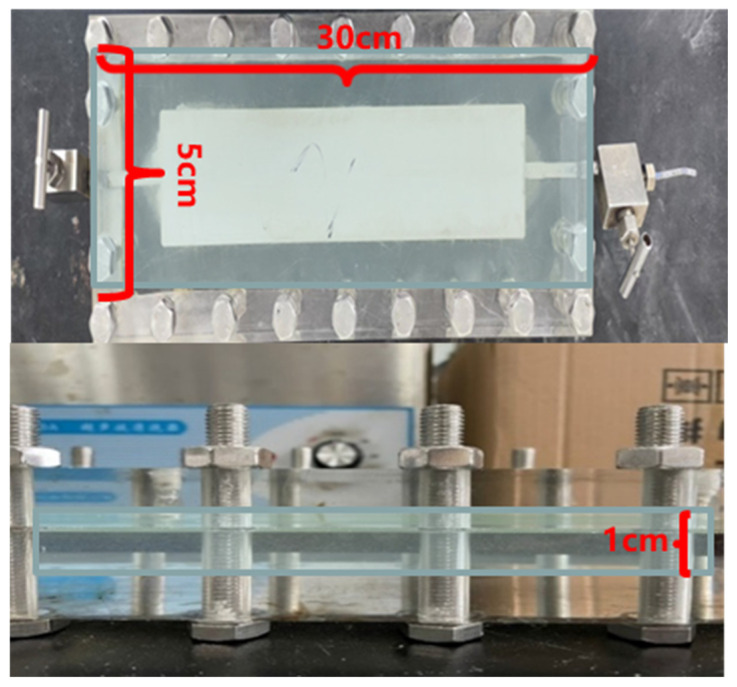
The specifications of the two-dimensional flat plate model.

**Figure 25:**
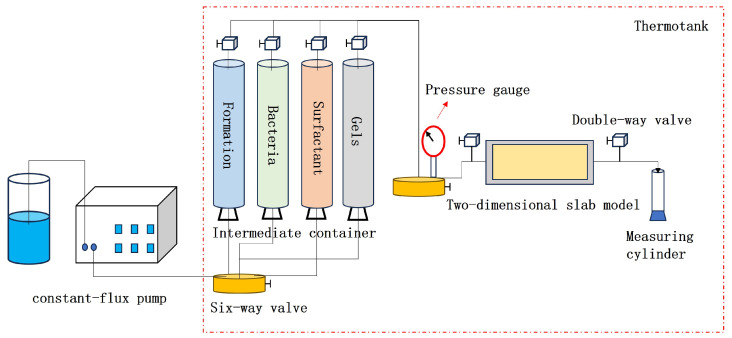
Schematic diagram of the two-dimensional slab model experimental setup.

A two-dimensional plate model of size 30 × 5 × 1 cm (Kg = 800 mD) was dried for 24 h, as shown in Figure 24. After weighing the dry weight, the model was vacuumed for 24 h and then saturated with formation water. After full saturation, the wet weight was measured to calculate the pore volume. After the saturated two-dimensional model underwent water flooding for 20 PV, the liquid permeability was calculated once the pressure stabilized. Next, the two-dimensional model was placed in a constant temperature box at 50 °C and saturated with crude oil at a rate of 0.1 mL/min until no water was discharged from the outlet. The volume of the saturated oil was recorded. After the core was aged for 7 days, water flooding was carried out at the optimal rate determined in Section 4.6.2 and it was continued until the water content at the outlet reached 98%. The changes in pressure and flow rate during the water flooding process were detected and recorded. For the water-flooded model, ICRG gel and formation water were injected in the same way as described in Section 4.6.3 and Section 4.6.4. After 1 day of coagulation, the optimized flooding oil system was injected. The valves at both ends of the two-dimensional model were closed. After 5 days of constant temperature culture, water flooding was continued until the water content reached 98%. Parameters such as microbial concentration, microbial metabolites, and community structure under different displacement ratios during the water flooding process were recorded. Crude oil components of the extracted oil samples were analyzed to evaluate the changes in crude oil composition before and after microbial action. The experimental setup is shown in Figure 25.

## Figures and Tables

**Table 1:** The NBCI comparison results of microbial samples.

ID No.	Number	DNA Identification Results	Identities
Z4067-1	ZY-1	Bacillus licheniformis	99.58%

**Table 2:** The changes in oil–water interfacial tension and crude oil viscosity before and after emulsification of crude oil.

Before Emulsification (50 °C)		After Emulsification (50 °C)	
Oil viscosity, mPa·s	Interfacial tension, mN/m	Oil viscosity, mPa·s	Interfacial tension, mN/m
1480	9.7	520	3.7

**Table 3:** The cross-linking and gelation of scleroglucan at different concentrations.

No.	Polymer	Cross-Linking Agent	Gelation Strength-24 h			Gelation Strength-48 h		
1	1%scleroglucan	1% phenolic resin	E	E	E	F	F	F
2	2%scleroglucan		F	F	F	G	G	H
3	3%scleroglucan		G	G	G	H	H	H

**Table 4:** Water displacement recovery rate at different displacement velocities (water content 98%).

Rock Sample	Permeability, mD	Porosity,%	Irreducible Water Saturation, %	Displacing Velocity, mL/min	Recovery, %
YX-1	765	27	24	0.1	14
YX-2	786	28	22	0.1	15
YX-3	785	30	21	0.3	21.9
YX-4	820	31	16	0.3	20.5
YX-5	785	31	18	0.5	18.9
YX-6	812	28	20	0.5	17.8
YX-7	820	26	24	1	12
YX-8	837	26	23	1	13

**Table 5:** Optimization of different oil displacement combination systems.

Schemes	Permeability (mD)	The System of Oil Displacement
1	800	Surfactant (0.5 PV)
2		Microorganisms (0.5 PV)
3		Mixed injection: Microorganisms + Surfactant (0.5 PV)
4		Blank control

**Table 6:** Combination optimization schemes for different oil displacement systems.

Number	The Combination of Oil Displacement System
1	Surfactant (0.5 PV)
2	Microorganism (0.5 PV)
3	Mixed injection: Microorganism+ Surfactant (0.5 PV)
4	Control group

**Table 7:** Displacement components under different displacement multiples.

	Initial	1-1	1-2	1-3	1-4	1-5	1-6
The water content/%	98.4	57.14	66.67	80.00	87.50	95.00	98.50
Displacement multiple/PV	2.73	2.76	2.84	2.88	2.9	2.99	3.2

**Table 8:** Optimization scheme for oil displacement system injection volume.

No.	Oil Displacement System (No.)	Injection Volume/PV	Number of Experiments
1	1, 2, 3, 4	0.3	2
2	1, 2, 3, 4	0.5	2
3	1, 2, 3, 4	0.6	2
4	1, 2, 3, 4	0.7	2

## Data Availability

The original contributions presented in this study are included in the article. Further inquiries can be directed to the corresponding authors.
